# Defining Roles and Responsibilities for School-Based Tele-Facilitators: Intraclass Correlation Coefficient (ICC) Ratings Of Proposed Competencies

**DOI:** 10.5195/ijt.2021.6351

**Published:** 2021-06-22

**Authors:** Hannah Douglass, J. Joneen Lowman, Vrushali Angadi

**Affiliations:** 1 University of Kentucky, Lexington, Kentucky, USA

**Keywords:** e-Helper, Facilitator, Schools, Telehealth, Telepractice, Training

## Abstract

The primary purpose of this study was to craft and validate a set of core competencies necessary for a tele-facilitator to possess in the school setting. Competencies were created through literature review and qualitative analysis. Following expert review, the competencies were revised and formatted into an online survey which was sent to respondents in four target groups: (a) school administrators who had adopted telepractice as a service delivery model; (b) speech-language pathologists (SLPs) experienced in telepractice within a school setting; (c) current tele-facilitators, and (d) scholars experienced in telepractice. Fifty-seven percent (20 out of 35) of the competencies were rated as “Essential Skills.” The remaining competencies could be more or less important depending on workplace requirements.

During the 2018-2019 school year, the primary disability of “speech-language impaired” was the second most prevalent disability among students receiving services under the Individuals with Disabilities Education Act, accounting for 19% of the total exceptional student education population (National Center for Education Statistics, 2020). Speech-language pathologists (SLPs) are the experts charged with addressing the communication needs of said population. SLP personnel shortages have plagued the schools for decades making telepractice an attractive option to school districts seeking to comply with federal mandates (American Speech-Language-Hearing Association (ASHA) Telepractice Key Issues, n.d.; ASHA Schools Survey Report: SLP Workforce and Work Conditions, 2016a; ASHA Schools Survey Report: SLP Workforce and Work Conditions Trends, 1995 – 2014). In a typical school-based telepractice model, the SLP and child connect through a real-time audio/video system. The child is monitored by a tele-facilitator, an adult who assists with the technology, manipulation of physical materials, and behavior management as directed by the SLP.

Tele-facilitators have been cited in the telehealth and telepractice literature as being integral to the success of a tele-program (Alvares, 2013; Grogan-Johnson, 2013; Waibel et al., 2017). As with any skilled job or profession, training for tele-facilitators is indicated to ensure client/patient safety, privacy, and satisfaction with the encounter. Furthermore, training has been recommended as integral to successful implementation of telehealth programs (Ross et al., 2016). Competencies are the foundation on which quality training is built. Baker and colleagues (2009) define competencies as the “skills and abilities employees are expected to have or develop, as well as the processes required to achieve success,” and provide employers a means to evaluate employee performance and monitor professional development. Despite the necessity for competencies, reviews of the literature reveal a dearth of research to support minimum knowledge and skills a competent tele-facilitator should possess across disciplines in telehealth (Coco et al., 2020; Edirippulige & Armfield 2017; Ross et al., 2016). Uncertainty regarding necessary skills is complicated by the lack of consistent nomenclature for tele-facilitators. Patient presenter is the term commonly used in telemedicine, while in the school setting e-helper or tele-facilitator is the preferred terminology (Alvares, 2012; Coco et al., 2020; Waibel et al., 2017). For the purposes of this article, the term tele-facilitator will be used.

The emergence of degree and certificate programs designed for tele-facilitators makes it evident that the need for training is being recognized, yet considerable variation exists amongst current training programs in title, degree type, and length/delivery of training. Little information is available regarding learning outcomes specific to each certificate program, making it unclear as to the exact competencies program graduates exit having acquired (International Board of Credentialing and Continuing Education Standards, 2020; National School of Applied Telehealth, n.d.; Thomas Jefferson University, 2019). Industry standards for required knowledge and skills have been developed by the American Telemedicine Association (2011), however, the standards are written for tele-facilitators delivering medical care to adult patients. To date, no guidelines have been developed for tele-facilitators assisting rehabilitation therapists treating pediatric clients across settings, much less in a school setting.

Per ASHA telepractice recommendations, the SLP is at least partially responsible for providing training to tele-facilitators (n.d.). Despite this responsibility, only 23% of SLPs responding to the ASHA SIG 18 Telepractice Survey felt they were “very prepared” to identify and train facilitators at the outset of their telepractice career, compared to those who responded “not at all prepared” (23%) and “somewhat prepared” (54%) (2016). Similarly, Tucker (2012) conducted qualitative interviews with five school-based SLPs experienced in telehealth, and found that untrained tele-facilitators resulted in major barriers to treatment delivery (“could not set up the equipment properly; did not get the students to therapy on time; could not effectively manage student behavior, and did not have therapy materials ready”) (p. 51).

The lack of definitive competencies further confounds the SLP's ability to properly train a tele-facilitator. The University of Maine has created a list of tele-facilitator competencies; however, these should be viewed as recommendations due to the lack of any formal validation (Perkins Walker, 2015). Other published guidelines describe minimum competencies required of a tele-facilitator working within a medical setting and/or interacting with an adult population (American Telemedicine Association, 2011; California Telehealth Resource Center, 2014; Great Plains Telehealth Resource & Assistance Center, 2012; Meyer et al., 2012). For example, Houwelingen et al. (2016) examined competencies required for telehealth activities specific to the field of nursing. Similarly, the American Telemedicine Association (2011) developed guidelines for training tele-facilitators, with most of the writing committee (17 out of 25 members) having backgrounds in nursing or medicine. Treating pediatrics within a school setting requires competencies not addressed in these resources such as knowledge of Individual Education Programs (IEPs), the Family Educational Rights and Privacy Act (FERPA), and behavior management. While the need for training resources is clearly being recognized, there continues to be a deficit in core competencies specific to the pediatric population in speech-language pathology.

Tele-facilitators are critical to the implementation of a successful telepractice program as these individuals are the in-person link between the speech-language pathologist and the child. Despite this, minimal attention has been given to the competencies required of tele-facilitators assisting pediatric populations within a school setting. Therefore, a need exists to identify a set of competencies that all educational tele-facilitators must possess to ensure children receive high quality speech-language services via telepractice. The purpose of this project was to develop a set of core competencies for tele-facilitators working in a school setting. Specifically, the project sought to:

1. Craft a set of core competencies reflecting minimum skills required of all school-based tele-facilitators assisting speech-language pathologists during telepractice.
2. Validate the set of core competencies by four user groups (school administration, SLPs experienced in telepractice, current e-helpers, and academics with expertise in telepractice).
3. Determine if differences exist among the four user groups’ perceptions of competencies required of school-based tele-facilitators.

## METHOD

The competency checklist was developed through a three-step process: literature review, expert review, and consumer review. The study was approved by the university IRB.

### STEP 1: LITERATURE REVIEW

A literature search was conducted across thirteen databases as well as a general search engine (Google). The original search was conducted through the year of 2017 and was updated in October 2019. Varying search terms related to tele-facilitator and training were used as detailed in Table 1. Searches ranged from zero to 460 results.

**Table 1:** Summary of Literature Search Strategy

Databases	Example Search Terms
EBSCOhost; PubMed; Cochrane Library; Academic Search Complete; AgeLine; CINAHL; Dentistry & Oral Sciences Source; ERIC; MEDLINE; Psychology and Behavioral Sciences Collection; PsycINFO; Google; ProQuest Science, Technology and Medical Combined Search; Abstracts in New Technology & Engineering	Telepractice; Telehealth; Telemedicine; Telecare; e-Helper; Training; Facilitator; Training; Telepresenter; Remote Site Coordinator; Telehealth Program Manager; Telemedicine; Helper

*Note.* From Schlaak, H. M., & Lowman, J. (2018). *Professional competencies for e-helpers: A telepractice resource*. Theses and Dissertations—Communication Sciences and Disorders. https://doi.org/10.13023/ETD.2018.110

### INCLUSION/EXCLUSION CRITERIA

Twenty-three articles and/or resources (henceforth referred to as “sources”) were selected for initial inclusion based on title and abstract screening. Sources were included if they addressed any aspect of skills or training required of the tele-facilitator. Any experimental design including systematic reviews and meta-analyses were included. Given the lack of peer reviewed or refereed sources, gray literature such as commercial organization and telehealth resource websites were included. Peer-reviewed articles and web-resources were excluded if training guidelines were limited to a specific technology or software, were not relevant to a tele-facilitator, or did not specify discrete skills or skill areas. No year limits were placed on the search to ensure all available evidence was included. However, the search was restricted to articles published in English.

After applying inclusion/exclusion criteria, nine of the 23 sources met the inclusion criteria. Fourteen of the 23 sources were rejected based on the exclusion criteria. The nine sources chosen based on the inclusion/exclusion criteria are detailed in Table 2. Four of the sources were applicable to the school setting. Five of the sources were medically or clinically geared, with two being specific to nursing. No new sources were found in the updated search.

**Table 2:** Included Resources

Title	Target Setting	Publication Type	Author(s), Year
Working with Facilitators to Provide School-Based Speech and Language Intervention via Telepractice.	Educational (school)	Peer-Reviewed Journal	Alvares, 2013
The CTRC Telehealth Program Developer Kit.	Clinical (nonspecific)	Industry Website	California Telehealth Resource Center, 2014
Telepresenter Competency Check List – (RN)	Medical (nursing)	Industry Website	Great Plains Telehealth Resource & Assistance Center, 2012
Teletherapy: Serving School-Age Children in K. T. Houston (Author), *Telepractice in Speech-Language Pathology*	Educational (school)	Book	Grogan-Johnson, 2014
Competencies required for nursing telehealth activities: A Delphi-study	Medical (nursing)	Peer-Reviewed Journal	Houwelingen et al., 2016
Expert Consensus Recommendations for Videoconferencing-Based Telepresenting	Clinical (nonspecific)	Industry Website	American Telemedicine Association, 2011
Essential Telemedicine Elements (Tele-Ments) for Connecting the Academic Health Center and Remote Community Providers to Enhance Patient Care	Medical (nonspecific)	Peer-Reviewed Journal	Meyer et al., 2012
Perspectives of Speech-Language Pathologists on the Use of Telepractice in Schools: The Qualitative View	Educational (school)	Peer-Reviewed Journal	Tucker, 2012
e-Helper Competencies	Variable (school, home, clinic)	University Website	Perkins Walker, 2015

*Note.* From Schlaak, H. M., & Lowman, J. (2018). *Professional competencies for e-helpers: A telepractice resource*. Theses and Dissertations–Communication Sciences and Disorders. https://doi.org/10.13023/ETD.2018.110

### QUALITATIVE ANALYSIS

Thematic analysis was utilized to analyze and compile the initial competencies (Braun & Clarke, 2006). The first author compiled competencies across the nine sources into a Microsoft Excel spreadsheet. The first author completed first level coding by identifying broad categories with keywords such as “privacy,” “administration,” and “troubleshooting.” Competencies were re-organized according to broad categories. The second author independently reviewed categorization and discrepancies in coding were resolved by discussion until consensus was reached. In second level coding, broad categories were further condensed and competencies irrelevant to the school setting were eliminated. Second level coding was completed independently by the first and second author. Consensus on coding was reached through discussion. “Universal” competencies were identified by examining the data for competencies that recurred across at least five of the nine sources. The resulting 34 competencies were revised to reflect the specific needs of a school setting and ensure consistent verbiage. For example, competencies referencing privacy and confidentiality were rewritten and standardized to specifically address FERPA.

The 34 competencies were then organized into the five identified thematic areas:

- **Telepractice Session** (12 competencies): Competencies referring to duties occurring during the telepractice session (e.g., helping the patient access web-based therapy tools).
- **Technology Specific Knowledge** (12 competencies): Competencies referring to operation and maintenance of technology (e.g., establishing and troubleshooting video/audio connection).
- **Interpersonal Skills** (3 competencies): Competencies referring to communication (e.g., increasing patient confidence in technology).
- **Policies and Procedures** (5 competencies): Competencies referring to organizational policies (e.g., ensuring HIPAA compliance).
- **Administrative Knowledge and Skills** (3 competencies): Competencies referring to record keeping and scheduling (e.g., entering patient information as needed).

### STRATEGIES FOR TRUSTWORTHINESS

There were two main techniques implemented to show trustworthiness in the formation of the school-based competencies: audit trails and peer debriefing sessions. Audit trails were used to document steps in the coding process and decision-making. Weekly peer debriefing sessions were conducted to discuss coding decisions and come to a consensus on coding.

### STEP 2: CONTENT EXPERT REVIEW

Content expert review was completed using an iterative process modeled after Grant and Davis (1997). Four content experts were recruited to review the initial competencies. Content experts represented diverse backgrounds of healthcare administration, direct service providers, and telehealth researchers. Content experts were asked to rate the importance of each competency relative to the role of a tele-facilitator on a 10-point Likert scale, with one being least important and 10 being most important. An average score was calculated for each competency across all content experts. Competencies with an average score of greater than five were included in the survey. No competencies had an average rating of less than five. Ninety-one percent of competencies (31 out of 34) were rated with average scores being greater than or equal to eight. The checklist was revised to reflect expert suggestions which included changes in verbiage, an additional competency, and revision of the Likert scale to a 5-point scale. The revised competency checklist had a total of 35 discrete competencies organized into five thematic areas.

### STEP 3: CONSUMER REVIEW

Survey participants were recruited from four consumer groups: (a) school administrators who had adopted telepractice as a service delivery model in their school; (b) SLPs experienced in telepractice within a school setting; (c) current school-based tele-facilitators, and (d) scholars engaged in telepractice at an institution of higher education. The rationale for choosing these groups was to provide a varied viewpoint that included direct and indirect service providers.

Consumer review was captured using the secure online software tool Qualtrics. The survey was organized into four sections: consent and survey directions, initial qualifying questions, competency rating scales, and participant demographics. To qualify for participation, the respondent had to self-identify as a member of one of the four consumer groups and have had direct or indirect experience with telepractice in a school setting. Following completion of survey consent, qualified participants were asked to rate the 35 individual competencies, which were organized by the thematic areas listed above. Participants were asked to rate the importance of each competency relative to the role of the tele-facilitator on a 5-point Likert scale with 1 (*not important*) to 5 (*very important*). At the end of each thematic area, participants had the option to make comments in a free-text box. Participant demographic questions varied based on the participants’ selected job positions. Questions were aimed at understanding the duration of experience with telepractice, settings, purposes, experience with training facilitators, and formal training in telepractice itself.

### SURVEY SAMPLING

The sampling method was a convenience sample of participants who were recruited from a variety of sources including: telehealth companies with a school-based division, school districts using telepractice, and the American Speech-Language Hearing Association's Special Interest Group 18, Telepractice. A university IRB approved email invitation to participate in the study was distributed through the aforementioned recruitment sources. Participants were encouraged to disseminate the anonymous survey link to other individuals in the four defined user groups. The survey was open for a total of eight weeks.

### QUANTITATIVE ANALYSIS

Survey ratings were analyzed using Intra-Class Correlation Coefficients (ICC). ICC estimates and their 95% confidence-intervals were calculated using SPSS statistical package version 23 (SPSS Inc., Chicago, IL) based on a mean-rating, absolute-agreement, two-way random-effects model. The ICC coefficients were interpreted based on the recommendations by (Koo & Li, 2016) in which ICC values of less than 0.5 are indicative of poor reliability, values between 0.5 and 0.75 indicate moderate reliability, values between 0.75 and 0.9 indicate good reliability, and values greater than 0.90 indicate excellent reliability. Participants were split between “Direct Providers” (speech-language pathologists and tele-facilitators) and “Indirect Providers” (school administrators and university faculty). Participants were divided based on the hypothesis that direct providers would have different priorities or preferences for essential skills than indirect providers, and to ascertain if differences in ratings existed between the two groups.

ICC values are a measurement of the level of agreement between raters and are therefore not a measure of quality of the product being rated. For example, if all 25 participants rated a given competency as “least important,” the ICC value would likely be “good” or “excellent” (ICC of .75-1.0), despite rating the quality of the competency as not important. Therefore, descriptive statistics (mean rating and standard deviation) were also calculated for each competency to measure quality.

## RESULTS

For the purpose of conciseness, the following abbreviations will be used henceforth with regard to survey respondents: Speech-language pathologists will be referred to as “SLPs;” tele-facilitators will be referred to as “TF;” school administrators will be referred to as “SA;” finally, university faculty/staff will be referred to as “UF.”

### PARTICIPANTS

A total of 21 SLPs completed the survey in its entirety; of those, 16 were currently using telepractice and five had used telepractice in an educational setting previously. The average experience in telepractice ranged from 1 month to 9.25 years. A total of three TF currently assisting with telepractice in an educational setting completed the survey, with average experience ranging from 2 months to 1.5 years. The lone SA indicated that the school district had contracted for telepractice services for 4 years. Seven UF completed the survey. Questions about years of experience and caseload size were not relevant to UF due to the domain of expertise being in research and/or other indirect telepractice services. See Table 3 for complete details of demographic data.

**Table 3:** Participant Demographic Settings

Settings	SLP	TF	SA	Total
	*n* = 21	*n* = 3	*n* = 1	*n* = 25
Preschool	7	2	0	9
Elementary	19	2	1	22
Secondary school (middle/high school)	16	2	0	18
Special day/residential school	0	0	0	0
College/university	0	N/a	N/a	0
Home	6	N/a	N/a	6
Other	0	0	0	0

*Note.* From Schlaak, H. M., & Lowman, J. (2018). *Professional competencies for e-helpers: A telepractice resource*. Theses and Dissertations–Communication Sciences and Disorders. https://doi.org/10.13023/ETD.2018.110

The majority of SLPs and TF indicated they used telepractice with students in elementary through high school grades. Fewer respondents across all SLPs and TF indicated they served children in preschool and other environments. Most SLPs reported using telepractice for assessment, treatment, IEP related activities, professional consultation, and parent conferences. The majority of SLPs indicated using telepractice to assess and/or treat articulation and/or phonological disorders and language disorders. All three TF indicated they assisted with treatment. The one SA reported telepractice used in an elementary school setting for assessment and treatment purposes. See Table 4 for complete details regarding telepractice usage.

**Table 4:** Participants’ Use of Telepractice

Purpose	SLP	TF	SA	Total
	*n* = 21	*n* = 3	*n* = 1	*n* = 25
Assessment	15	2	1	18
Treatment	21	3	1	25
Follow-up/monitoring	9	N/a	N/a	9
Professional consultation (i.e., with teachers, administrators, other related services)	16	2	N/a	18
Individualized Education Plan (IEP) related activities	18	1	1	20
Parent conferences	15	0	0	15
Other	2	0	0	2

*Note.* From Schlaak, H. M., & Lowman, J. (2018). *Professional competencies for e-helpers: A telepractice resource*. Theses and Dissertations--Communication Sciences and Disorders. https://doi.org/10.13023/ETD.2018.110

### TELE-FACILITATOR TRAINING

Sixty-six percent of SLPs reported being responsible for training a facilitator, of these, 64% of SLPs indicated providing informal training simultaneously with delivery of services. All TF indicated receiving training by self-guided learning and/or informal peer-to-peer mentoring. No TF indicated having received any formal training. The SA indicated training of facilitators was conducted by “other” but did not elaborate. Seventy-one percent of UF indicated responsibility for training facilitators. Seventy-one percent of UF indicated informal training simultaneously with delivery of services, while 43% of UF indicated formal training via videoconferencing.

### INTER-RATER RELIABILITY OF COMPETENCIES

ICC estimates and their 95% confidence-intervals were calculated based on a mean-rating, absolute-agreement, two-way random-effects model for the Direct Providers (SLP/TF) group, the Indirect Providers (UF/SA) group, and for the group as a whole (Direct + Indirect Providers combined). ICC values were interpreted according to Koo and Li (2016). ICC ratings will be discussed by thematic area, and then as a whole checklist. Complete ICC results for Direct Providers are reported in Table 5. Indirect Provider ICC results are reported in Table 6. Whole group ICC values are reported in Table 7.

**Table 5:** Direct Providers ICC Calculation in SPSS Using Single-Rating, Absolute-Agreement, 2-Way Random-Effects Model, Average Measures

	Intraclass Correlation	CI_95%_		F Test with True Value 0			
		Lower Bound	Upper Bound	Value	df1	df2	Sig
Telepractice Session	.863^a^	0.771	0.93	7.322	25	275	0
Technology	.782^a^	0.636	0.888	5.45	24	264	0
Interpersonal	.598^a^	0.248	0.805	2.697	24	48	0.002
Policy/Procedure	0.672^a^	0.424	0.836	3.234	24	96	0
Administrative	.781^a^	0.575	0.897	4.576	24	48	0
All	.887^a^	0.81	0.94	12.47	23.00	782.00	0.00

*Note.* CI= confidence interval; Sig= significance

a This estimate is computed assuming the interaction effect is absent, because it is not estimable otherwise.

**Table 6:** Indirect Providers ICC Calculation in SPSS Using Single-Rating, Absolute-Agreement, 2-Way Random-Effects Model, Average Measures

	Intraclass Correlation	CI_95%_		F Test with True Value 0			
		Lower Bound	Upper Bound	Value	df1	df2	Sig
Telepractice Session	.658^a^	0.11	0.931	2.927	6	66	0.014
Technology	.579^a^	0.082	0.894	2.711	7	77	0.014
Interpersonal	.582^a^	−0.106	0.9	2.909	7	14	0.042
Policy/Procedure	.174^a^	−0.898	0.793	1.243	7	28	0.314
Administrative	.937^a^	0.787	0.986	15.85	7	14	0.000
All	.737^a^	0.427	0.942	5.376	6	204	0.000

*Note.* CI= confidence interval; Sig= significance

a This estimate is computed assuming the interaction effect is absent, because it is not estimable otherwise.

**Table 7:** Whole Group ICC Calculation in SPSS Using Single-Rating, Absolute-Agreement, 2-Way Random-Effects Model, Average Measures

	Intraclass Correlation	CI_95%_		F Test with True Value 0			
		Lower Bound	Upper Bound	Value	df1	df2	Sig
Telepractice Session	0.766	0.621	0.869	6.3	32	352	0.000
Technology Specific	0.744	0.599	0.854	4.625	32	352	0.000
Interpersonal Skills	0.631	0.349	0.804	3.092	32	64	0.000
Policies/Procedures	0.591	0.337	0.772	2.645	32	128	0.000
Administrative	0.793	0.587	0.897	6.162	32	64	0.000
All	0.87	0.8	0.929	11.066	30	1020	0.000

*Note.* CI= confidence interval; Sig= significance

For the Direct Providers group, ICC values for each thematic grouping ranged from .598 to .863 (“moderate” to “good” reliability), with Interpersonal competencies having the lowest value and Telepractice Session competencies having the highest value. For the checklist as a whole, the Direct Providers group had an ICC of .887 (“good” reliability). See Table 5 for complete details. For the Indirect Providers group, ICC values for each thematic grouping ranged from .174 to .937 (“poor” to “excellent” reliability), with Policy/Procedure competencies having the lowest value and Administrative competencies having the highest value. For the checklist as a whole, the Indirect Providers group had an ICC of .737 (“moderate” reliability). See Table 6 for complete ICC results for the Indirect Providers group. For all respondents, ICC values for each thematic grouping ranged from .591 (“moderate” reliability) to .793 (“good” reliability), with Policies/Procedure competencies having the lowest value and Administrative competencies have the highest value. To determine how all respondents rated the entire checklist, overall ICC was calculated for both groups with a value of .873 (“good” reliability). Complete details can be seen in Table 7.

### DESCRIPTIVE STATISTICS

To determine the perceived importance of the individual competencies across consumer groups, mean ratings from the Likert scale (1 = *least important* to 5 = *most important*) were rounded to the nearest tenth and compared and categorized as follows:

- Essential Skills – competencies with whole group means of 4.5–5
- Supplementary Skills – competencies with whole group means of 3.5–4.4
- Optional Skills – competencies with whole group means of 3.4 or less

Results will be discussed by skill category. For greater visual ease, competencies were shortened to key words to simplify the presentation in tables. Complete wording for competencies can be found in Appendix A.

### ESSENTIAL SKILLS

Of the 35 competencies, a total of 20 (57%) had a whole group mean rating between 4.5 and 5. Of the thematic areas, technology specific had seven competencies in Essential Skills, the most of all the thematic areas. All five of the competencies in the policy and procedures thematic area were included in the Essential Skills category. In the remaining thematic areas, telepractice session had six competencies, interpersonal had two competencies, and administration had no competencies in the Essential Skills category. For complete details, please see Table 8.

**Table 8:** Essential Skills Descriptive Statistics

		All Groups (SLP, TF, SA, UF)	Direct Providers (SLP, TF)	Indirect Providers (SA, UF)
Telepractice Session Competencies				
Verifies Technology	*n*	35.00	27.00	8.00
	*M*	5.00	5.00	5.00
	*SD*	0.00	0.00	0.00
Positions Technology	*n*	35.00	27.00	8.00
	*M*	4.94	4.93	5.00
	*SD*	0.34	0.39	0.00
Ensures Audio/Visual quality	*n*	35.00	27.00	8.00
	*M*	4.83	4.78	5.00
	*SD*	0.75	0.85	0.00
Escorts Students	*n*	35.00	27.00	8.00
	*M*	4.71	4.85	4.25
	*SD*	0.71	0.53	1.04
Web-Based Therapeutic Materials	*n*	35.00	27.00	8.00
	*M*	4.71	4.63	5.00
	*SD*	0.99	1.12	0.00
Behavior Management	*n*	33.00	26.00	7.00
	*M*	4.52	4.62	4.14
	*SD*	1.00	0.80	1.57
Technology Specific Competencies				
Accesses Session Link	*n*	34.00	26.00	8.00
	*M*	4.94	4.92	5.00
	*SD*	0.34	0.39	0.00
Establishes Session Link	*n*	34.00	26.00	8.00
	*M*	4.94	4.92	5.00
	*SD*	0.34	0.39	0.00
Troubleshoots Audio/Video	*n*	34.00	26.00	8.00
	*M*	4.59	4.62	4.50
	*SD*	1.08	0.98	1.41
Troubleshoots Software	*n*	34.00	26.00	8.00
	*M*	4.47	4.54	4.25
	*SD*	1.13	1.03	1.49
Communicates with SLP	*n*	34.00	26.00	8.00
	*M*	4.88	4.92	4.75
	*SD*	0.48	0.39	0.71
Troubleshooting Resource List	*n*	34.00	26.00	8.00
	*M*	4.71	4.62	5.00
	*SD*	0.72	0.80	0.00
Communicates with IT	*n*	33.00	25.00	8.00
	*M*	4.82	4.76	5.00
	*SD*	0.58	0.66	0.00
Policy and Procedure Competencies				
General School Policies	*n*	33.00	25.00	8.00
	*M*	4.88	4.84	5.00
	*SD*	0.49	0.55	0.00
School Privacy Policies	*n*	33.00	25.00	8.00
	*M*	5.00	5.00	5.00
	*SD*	0.00	0.00	0.00
Infection Control Policies	*n*	33.00	25.00	8.00
	*M*	4.88	4.92	4.75
	*SD*	0.49	0.40	0.71
School Telepractice Policies	*n*	33.00	25.00	8.00
	*M*	5.00	5.00	5.00
	*SD*	0.00	0.00	0.00
Privacy Policies within Telepractice	*n*	33.00	25.00	8.00
	*M*	5.00	5.00	5.00
	*SD*	0.00	0.00	0.00
Interpersonal Competencies				
Student Interaction	*n*	33.00	25.00	8.00
	*M*	4.88	5.00	4.50
	*SD*	0.46	0.00	0.93
Facilitates Communication	*n*	33.00	25.00	8.00
	*M*	4.82	4.92	4.50
	*SD*	0.58	0.40	0.93

*Note.* For simplification of visual presentation, competencies were shortened to key words. Complete competency descriptors can be found in Appendix A.

### SUPPLEMENTARY SKILLS

Of the 35 competencies, a total of 12 (34%) had a whole group mean rating between 3.5 – 4.4. Of the thematic areas, “technology specific” had five competencies in Supplementary Skills, the most of all the thematic areas. In the remaining thematic areas, “telepractice session” had four competencies, “administrative” had two competencies, and “interpersonal” had one competency. “Policy and procedure” had no competencies in this category. For complete details, please see Table 9.

**Table 9:** Supplementary Skills Descriptive Statistics

		All Groups (SLP, TF, SA, UF)	Direct Providers (SLP, TF)	Indirect Providers (SA, UF)
Telepractice Session Competencies				
Therapeutic Materials	*n*	35.00	27.00	8.00
	*M*	4.31	4.41	4.00
	*SD*	1.28	1.22	1.51
Clean Equipment	*n*	35.00	27.00	8.00
	*M*	3.80	3.74	4.00
	*SD*	1.39	1.38	1.51
Clarify Responses	*n*	35.00	27.00	8.00
	*M*	3.51	3.37	4.00
	*SD*	1.77	1.84	1.51
Therapeutic Assistance	*n*	33.00	26.00	7.00
	*M*	3.79	3.85	3.57
	*SD*	1.58	1.62	1.51
Technology Specific Competencies				
Annotation Features	*n*	34.00	26.00	8.00
	*M*	3.88	3.92	3.75
	*SD*	1.49	1.62	1.04
Chat Feature	*n*	34.00	26.00	8.00
	*M*	4.06	4.38	3.00
	*SD*	1.41	1.24	1.51
Troubleshoots Peripherals	*n*	34.00	26.00	8.00
	*M*	3.76	3.77	3.75
	*SD*	1.48	1.51	1.49
Troubleshooting Log	*n*	33.00	25.00	8.00
	*M*	4.03	4.04	4.00
	*SD*	1.43	1.43	1.51
Account Management	*n*	33.00	25.00	8.00
	*M*	4.33	4.36	4.25
	*SD*	1.29	1.25	1.49
Administrative Competencies				
Manages IEP Paperwork	*n*	33.00	25.00	8.00
	*M*	4.21	4.68	2.75
	*SD*	1.41	0.95	1.67
Scheduling Therapy Sessions	*n*	33.00	25.00	8.00
	*M*	3.91	4.04	3.50
	*SD*	1.51	1.43	1.77

*Note.* For simplification of visual presentation, competencies were shortened to key words. Complete competency descriptors can be found in Appendix A.

### OPTIONAL SKILLS

Of the 35 competencies, a total of three (8%) had a whole group mean rating of 3.4 or less. Two of the competencies were in the thematic area of “telepractice session,” and were related to the facilitator's responsibilities for communicating with the SLP and students about homework and reminding students of assigned homework. The remaining competency was in the thematic area of “administrative,” and was related to the facilitator scheduling meetings between the SLP and others. For complete details, please see Table 10.

**Table 10:** Optional Skills Descriptive Statistics

		All Groups (SLP, TF, SA, UF)	Direct Providers (SLP, TF)	Indirect Providers (SA, UF)
Telepractice Session Competencies				
Homework Reminders	*n*	35.00	27.00	8.00
	*M*	2.49	2.63	2.00
	*SD*	1.56	1.67	1.07
Communicates with SLP about Homework	*n*	33.00	26.00	7.00
	*M*	3.36	3.54	2.71
	*SD*	1.45	1.45	1.38
Administrative Competencies				
Scheduling Meetings	*n*	33.00	25.00	8.00
	*M*	3.12	3.32	2.50
	*SD*	1.65	1.60	1.77
Interpersonal Competencies				
Describes Telepractice	*n*	33.00	25.00	8.00
	*M*	4.09	4.44	3.00
	*SD*	1.42	1.08	1.85

*Note.* For simplification of visual presentation, competencies were shortened to key words. Complete competency descriptors can be found in Appendix A.

### OPEN-ENDED COMMENTS

Respondents were given the option to make comments on any of the competencies at the end of each thematic area. A total of 17 comments were made throughout the survey by SLPs (n= 9) and UF (n=4). Three SLPs indicated that responsibilities such as maintaining homework, prompting during a therapy session, and scheduling meetings are not required and/or permitted of the facilitators in their work setting, or indicated scheduling responsibilities would be more appropriate for the SLP to assume. Conversely, one SLP indicated that delegating the scheduling of meetings and “paperwork flow” to the facilitator allowed for more students to be seen. A total of three comments were made relating to the importance of confidentiality. One SLP indicated that the tele-SLP should “provide a list of confidentiality expectations and how they should be handled” due to additional considerations in comparison to the average on-site position.

With regard to the facilitator's responsibility to answer clinical questions and describe telepractice accurately, one respondent, a UF, indicated that the facilitator should be careful to refer any clinical questions regarding student progress or outcomes to the SLP; while another UF indicated that facilitators should not be responsible for describing telepractice due to inaccurate descriptions. Finally, one SLP indicated that identification of a facilitator has been a “huge roadblock” in the success of some schools participating in telepractice and schools with designated facilitators are “significantly more successful.” Further, the same respondent indicated that there must be “complete buy-in” from the facilitator for the position to be successful.

## DISCUSSION

The purpose of this study was to fill a gap in the literature by providing the first evidence-based and formally validated set of competencies for tele-facilitators in the school setting. Providing this resource to SLPs and tele-facilitators is of vital importance to ensure children are receiving high quality services in a school setting. We accomplished our aims through a process of literature review, expert rating, and consumer rating. Consumers were recruited from variety of roles (SLP, TF, UF, SA) to determine if setting and/or role influenced competency ratings. Results will be discussed by the thematic area as well as a discussion of the competencies as a whole group.

### TELEPRACTICE SESSION COMPETENCIES

Half of the competencies in the thematic area of telepractice session were included in the Essential Skills category. Respondents valued competencies such as manipulation of technology during the session, escorting students to and from therapy, and behavior management. Competencies such as prompting/scaffolding during the session, clarifying student responses, and cleaning equipment between students fell into the Supplementary Skills category. Across groups, respondents did not tend to value the tele-facilitator following up on student's assigned homework. ICC ratings for this area were higher for the direct providers, indicating that indirect providers had more disagreement when it came to rating telepractice session competencies. Discrepancies in agreement and valuation may be explained by a difference in work settings. For example, some private tele-therapy companies may have protocols that prohibit tele-facilitators from prompting students or assigning homework.

### TECHNOLOGY SPECIFIC COMPETENCIES

Respondents generally valued technology related competencies, with seven of the 12 competencies appearing in the Essential Skills category. Highly rated competencies focused on troubleshooting and problem-solving technological difficulties, using basic features such as email and the internet, and managing accounts and privacy controls. These findings align with previous work citing the tele-facilitator's inability to troubleshoot technology problems as a primary barrier to the success of telepractice in a school setting (Alvares, 2013; Tucker, 2012). Lower rated competencies such as using annotation features and solving problems on local peripheral devices may be more an artifact of the respondent groups than the competencies’ importance. Direct Providers’ mean ratings of competencies such as proficiency in annotation features, account management, and troubleshooting peripheral devices were consistently higher than Indirect Providers’ ratings. Ratings could also be influenced by respondents’ lack of awareness and/or use of these features. This discrepancy between groups is mirrored in the ICC values. ICC values were higher for direct providers in this thematic area (ICC .782) indicating good reliability, while indirect providers fell into the moderate reliability range (ICC .579). These differences in agreement and mean ratings may be related to the lack of practical experience that indirect providers may have, leading to uncertainty in what technical skills are necessary for the tele-facilitator.

### INTERPERSONAL COMPETENCIES

Two of the three competencies in this thematic area were included in the Essential Skills category, indicating respondents valued the tele-facilitator's use of developmentally appropriate practices with students and accurate transmittal of information among personnel. ICC ratings for this thematic area were comparable across groups, meaning respondents rated interpersonal competencies similarly. A comparatively lower rated competency that should be highlighted is “[tele-facilitator] accurately describes telepractice to students, teachers, parents, administrators and or other professionals.” Though direct providers rated this competency as supplementary, indirect providers appeared to devalue this competency as optional. One university faculty respondent commented that tele-facilitators should never be responsible for describing telepractice to others because “they [often] describe it incorrectly or indicate it does not work because they are not comfortable with technology.” Conversely, one SLP stated “complete buy-in” from the tele-facilitator is necessary for the program to be successful.

The responsibility of tele-facilitators to accurately describe telepractice and its efficacy is heavily supported by the literature. Several authors have labeled them as key stakeholders, serving as “the face of the telepractice program” (Alvares, 2013; Tucker, 2012; Ross et al., 2016). As such, it is imperative that tele-facilitators receive adequate training to allow for confidence in their professional abilities as well as confidence in telepractice as an efficacious service delivery model. The discrepancy in responses regarding the tele-facilitator's responsibility to describe telepractice could be the result of the difference in use of telepractice. One hundred percent of university faculty reported conducting research in telepractice, while only 47% reported delivering clinical services via telepractice. Because the burden of collecting informed consent as part of the research process rests on study personnel, university faculty may lack understanding of the importance of the facilitator as a champion for telepractice.

### POLICY AND PROCEDURE COMPETENCIES

All respondents consistently rated competencies related to Policy and Procedures as the highest out of all competencies, with all five competencies appearing in the Essential Skills category. Three of the five competencies had a mean rating of 5. Respondents were adamant that the facilitator must adhere not only to school setting policies and procedures but also to telepractice procedures with heavy importance placed on confidentiality. While respondents valued the importance of telepractice policies, 71% percent of respondents indicated an absence of telepractice policies or awareness of such policies in their respective work settings. This is disconcerting, as the lack of telepractice policies and procedures has been identified as a barrier to successful telepractice programming (Tucker, 2012).

Interestingly, this thematic area was one of the lowest ICC ratings across groups, with poor reliability in the indirect provider group and moderate reliability in the direct provider group. The whole group ICC indicated moderate reliability. It is important to note that the accuracy of quantitative statistical analyses, such as ICCs, are largely dependent on sample size and sample variability. When there is little to no variability in the sample, the ICC is a less reliable measure (Mehta, 2018). A combination of these factors might explain the low ICC obtained in this thematic area. As a result, our inferences reflect an amalgamation of the qualitative and quantitative outcomes of the study. To this end, we recommend future researchers to adopt a holistic and comprehensive approach to data interpretation.

### ADMINISTRATIVE COMPETENCIES

The three competencies related to administrative skills had the lowest mean ratings of all 35 competencies, with no competency having a mean rating of greater than 4.5. However, direct providers had higher mean ratings than indirect providers on all three administrative competencies. For example, direct providers believed a tele-facilitator organizing IEP specific documentation was essential whereas indirect providers viewed it as optional. Respondents viewed competencies related to scheduling of meetings and therapy sessions as supplementary or optional. There was generally good agreement across groups for this thematic area, with direct providers having slightly less agreement than indirect providers.

Work setting could have influenced perceived importance of the tele-facilitator assisting with administrative duties. For example, private tele-therapy companies may have a system that eliminates the need for a facilitator to assist with scheduling, paperwork, and other administrative functions. While many survey comments indicated that scheduling should be completed by the SLP, one SLP respondent indicated that “the online therapy program is significantly more successful [when the facilitator] is able to […] assist the online therapist with onsite responsibilities such as coordinating meetings.” Similarly, another SLP respondent indicated that “having the facilitator help with scheduling meetings and helping with the flow of paperwork allowed me to see more students.” Discrepancies in work settings could also account for the difference in ICC ratings. Indirect providers may not have had a company policy in mind, leading to more agreement on what administrative duties should ideally be the tele-facilitator's responsibility.

### WHOLE CHECKLIST

For the checklist as a whole and its individual parts, indirect providers generally had less agreement than did direct providers. The exception to this was the administrative thematic area, in which indirect providers had an ICC value of .937 (excellent reliability) compared to .781 (moderate reliability) for direct providers. The generally lower reliability scores for the indirect providers group indicates that they did not tend to agree as much as the direct providers on the importance of a competency relative to the role of a tele-facilitator. As a whole rater group, the entire competency checklist ICC value indicated good reliability.

### TRAINING PRACTICES

The current study sought to identify minimum competencies that school-based facilitators should possess for the purpose of ensuring quality telepractice services. Competencies often serve as the building blocks for training. As part of the study, the PI and co-PI had a cursory level of interest in current training practices for the facilitator. While the sample size is small, 66% of SLPs reported being responsible for training a facilitator, yet no facilitators indicated having received training by a SLP. Further, 64% of SLPs and 71% of UF indicated training occurred informally in conjunction with the delivery of services.

Though exact demographic data for tele-facilitators across settings is unknown, data suggests that tele-facilitators may have diverse primary job titles such as paraeducator, principal, librarian, or parent (Coco et al., 2020; J. Tucker, personal communication April 10, 2017). Survey findings align with research indicating a lack of formal job-related training for paraprofessionals (Zobell & Hwang, 2020). When basic job training for paraprofessionals is inadequate, it is unsurprising that training for telepractice would be given priority. All parties involved in the telepractice session are negatively impacted when training occurs informally with the delivery of services. The client receives an inferior level of services, the facilitator lacks high quality personnel development, and the SLP is left attempting to balance therapeutic delivery with impromptu training.

The gap in training for facilitators could also be a result of the SLP not receiving adequate training in telepractice themselves. In ASHA's SIG 18 survey, the majority of SLPs (58%) report receiving their initial training from an employer/workplace, followed by 39% who received training from networking with colleagues, 35% from ASHA's practice portal on telepractice, and 34% from continuing education of 1-3 hours. Though these resources may be excellent sources of information, they are not synonymous with the more systematic and thorough education that graduate courses or continuing education workshops can provide. Only five percent of SLPs indicated having received any type of graduate-level courses in telehealth, while 25% of SLPs indicated attending continuing education workshops lasting one or more days. This inconsistency in telepractice training for the SLPs themselves could be creating a “trickle-down” effect whereby the SLP may not be aware of the importance of training the facilitator and/or lack the skills and confidence to provide adequate training.

### LIMITATIONS

This study was limited by the small sample size across respondent groups. In particular, the facilitators, school administrators, and university faculty groups were significantly smaller than the SLP group. Several reasons may account for the small sample size of facilitators. Many SLPs indicated that their tele-facilitators lacked sufficient time to take the survey due to their multiple responsibilities across the school day. Another factor could be that many tele-facilitators may have other titles as their primary role, and thus did not identify themselves as tele-facilitators. Other respondent groups, such as school administrators and university faculty, were difficult to identify. Study outcomes also could be limited by unclear survey instructions. Respondents were asked to rate competencies relative to their importance to the role of the tele-facilitator. Therefore, respondents may have interpreted a necessity to rate some competencies lower than others. Further, respondents may have allowed workplace policy to subjectively influence their ratings. For instance, respondents could have rated certain competencies as less important than others because it is not allowed in their specific company, rather than thinking of the checklist as a “gold standard” of preparedness.

## CONCLUSION

The identified competencies in the current study are a first step in providing SLPs with guidelines for the training of tele-facilitators. Survey respondents agreed upon the vast majority of competencies, while acknowledging that some competencies should be driven by specific work setting policies and procedures. Most discrepancies in agreement or valuation are likely a product of the differences in respondent roles or work settings. Therefore, it is imperative that future research include a diverse range of participants when examining outcome related measures for tele-facilitators. Regardless, the overall checklist had good reliability. As such, speech-language pathologists, school administrators, and university training programs can have confidence in the importance and relevance of the 20 competencies identified as Essential Skills to the roles and responsibilities of the tele-facilitator in a school setting.

## APPENDIX

*Full List of Competencies Divided by Thematic Area*

### Telepractice Session Competencies

- Verifies that all necessary technology is available for use during a telepractice session.
- Positions technology in relation to the student to maximize audio and video quality.
- Ensures lighting and noise levels are appropriate and will not interfere with audio and video quality.
- Assists student in accessing or using web-based therapy tools if necessary (e.g., highlighter, pointer, text tools, websites).
- Escorts the student to and from class if necessary
- Uses behavior management strategies as directed by the SLP.
- Prepares, organizes, and maintains therapeutic materials for use during the session (as directed by the SLP).
- Administers prompts, scaffolding and reinforcement as directed by the SLP.
- Clarifies student's response if necessary.
- Cleans equipment between students.

### Technology Specific Competencies

- Can access email and Internet to locate the links and online connections for the telepractice session.
- Can establish the video and audio connection for the telepractice session.
- Uses an alternative method to communicate with the SLP when video connection fails (e.g., telephone, email).
- Follows-up with technical support immediately following a therapy session if any problems occur.
- Solves audio and video problems on local electronic device (computer, tablet, etc.)
- Maintains a list of resources for assisting with troubleshooting technical problems (e.g., school personnel, video-conferencing platform, web resources etc.)
- Solves problems with software on local computer/tablet (e.g., loss of mouse, program not responding).
- Demonstrates ability to manage accounts, personal settings, and privacy controls in online telepractice application.
- Can use chat feature of video-conferencing software to communicate with SLP.
- Maintains a log of technical difficulties encountered and requests assistance with recurring problems.
- Can use annotation features of video-conferencing software (e.g., highlighter, arrow, text boxes).
- Solves problems with peripheral devices on local computer/tablet (e.g. external camera, printer, document camera).

### Interpersonal Competencies

- Interacts with students in a developmentally appropriate manner.
- Accurately transmits information between the SLP and students, teachers, parents, administrators, and or other professionals.
- Accurately describes telepractice to students, teachers, parents, administrators and or other professionals.

### Policy and Procedural Competencies

- Adheres to school district policy and procedures regarding telepractice (e.g., scheduling, roles, and responsibilities of a facilitator)
- Adheres to school district policy regarding student confidentiality (e.g., paperwork, electronic records, protected information in public spaces.)
- Adheres to school district policy specific to privacy and confidentiality within telepractice (e.g., video and audio privacy during tele-session.)
- Follows school's general operating policies and procedures (e.g., fire drill, emergency procedures, dismissal, lunch etc.)
- Follows school's infection control policies.

### Administrative Competencies

- Copies, sends, and collects paperwork specific to the Individualized Education Plan (IEP) process (e.g., consent to evaluate, annual IEP meetings).
- Assists with scheduling therapy sessions and make-up sessions as required.
- Schedules meetings between the SLP and families, teachers, and or additional professionals as warranted.
